# Incidence of Nontuberculous Mycobacterial Pulmonary Infection, by Ethnic Group, Hawaii, USA, 2005–2019

**DOI:** 10.3201/eid2808.212375

**Published:** 2022-08

**Authors:** Rebekah A. Blakney, Emily E. Ricotta, Timothy B. Frankland, Stacey Honda, Adrian Zelazny, Katrin D. Mayer-Barber, Samantha G. Dean, Dean Follmann, Kenneth N. Olivier, Yihe G. Daida, D. Rebecca Prevots

**Affiliations:** National Institutes of Health, Bethesda, Maryland, USA (R.A. Blakney, E.E. Ricotta, A. Zelazny, K.D. Mayer-Barber, S.G. Dean, D. Follmann, K.N. Olivier, D.R. Prevots);; Kaiser Permanente Hawaii, Honolulu, Hawaii, USA (T.B. Frankland, S. Honda, Y.G. Daida)

**Keywords:** Nontuberculous mycobacteria, Mycobacterium infections, nontuberculous, Mycobacterium avium complex, tuberculosis and other mycobacteria, ethnic group, Hawaii, United States, NTM, bacteria, respiratory infections

## Abstract

To further clarify differences in the risk for nontuberculous mycobacterial pulmonary infection (NTM-PI) among ethnic populations in Hawaii, USA, we conducted a retrospective cohort study among beneficiaries of Kaiser Permanente Hawaii (KPH). We abstracted demographic, socioeconomic, clinical, and microbiological data from KPH electronic health records for 2005–2019. An NTM-PI case-patient was defined as a person from whom >1 NTM pulmonary isolate was obtained. We performed Cox proportional hazards regression to estimate incidence of NTM-PI while controlling for confounders. Across ethnic groups, risk for NTM-PI was higher among persons who were underweight (body mass index [BMI] <18.5 kg/m^2^). Among beneficiaries who self-identified as any Asian ethnicity, risk for incident NTM-PI was increased by 30%. Low BMI may increase susceptibility to NTM-PI, and risk may be higher for persons who self-identify as Asian, independent of BMI.

In the United States, studies have indicated that risk for nontuberculous mycobacterial pulmonary disease (NTM-PD) differs by geographic location and ethnic group (*1*). Nontuberculous mycobacteria are environmental bacteria that are widespread in soil and water and can be acquired through the natural or built environment (*2*). Nationally, disease prevalence is highest in the southeastern United States and Hawaii (*1*,*3*), and disease is associated with selected environmental conditions (*2*), as well as with higher mycobacterial abundance in household plumbing (*4*). However, independent of geographic region, estimated prevalence is 2-fold higher among persons who self-identify as Asian/Pacific Islander than among those who self-identify as White (*1*). The incidence and prevalence of NTM infection and disease are increasing in the United States (*5*), and testing and positivity rates are highest among persons who self-identify as Asian (*6*); prevalence is also increasing in Hawaii (*7*) and the US-affiliated Pacific Islands (*8*).

Persons classified as Asian/Pacific Islander represent diverse populations, and aggregating these subpopulations may mask substantial heterogeneity (*9*). In a prior study in Hawaii, we identified substantial disparities in NTM pulmonary infection (NTM-PI) risk within Asian and Native Hawaiian and Other Pacific Islander (NHOPI) populations (*7*). To clarify ethnic disparities in NTM-PI risk, and particularly the role of BMI and other potential confounding factors among Asian/Pacific Islander populations, we conducted a retrospective cohort study among Kaiser Permanente Hawaii (KPH) beneficiaries. This research was approved by the KPH Institutional Review Board and was classified as nonhuman subjects research by the National Institutes of Health Office of Human Subjects Research Protection.

## Methods

### Study Population

We abstracted demographic, clinical, and microbiological data from KPH electronic health records (EHRs) for the years 2005–2019. We included beneficiaries for each year in which they were enrolled for >9 months; we excluded persons >90 years of age according to limited dataset regulations. Longitudinal demographic data included age on July 1 for each study year, sex, postal code, and census tract–level socioeconomic measures (median household income, neighborhood deprivation index, percentage graduated from high school). The neighborhood deprivation index is a measure of neighborhood-level socioeconomic status calculated by Kaiser Permanente (*10*). The index captures census tract–level measures of income, education, employment, housing, and occupation; higher scores indicate higher deprivation.

Demographic data included self-reported ethnicity; the standard KPH beneficiary enrollment form enables identification with >1 of 28 ethnic groups (Table 1). Additional health variables abstracted included height, weight, BMI, and smoking status. We searched EHRs for a set of prespecified codes from the International Classification of Disease, 9th and 10th revisions (ICD-9/10), for relevant underlying conditions ever coded. Although KPH does not serve the entire population of Hawaii, comparison with census data indicates that the KPH beneficiary population is generally representative of Hawaii residents with respect to age, ethnicity, and socioeconomic status (Appendix Table 2 ) (*11*,*12*). 

**Table 1:** NTM-PI incidence among Kaiser Permanente Hawaii beneficiaries, by ethnic group, Hawaii, USA, 2005–2019*

Ethnicity†	Beneficiaries, %	No. cases	Incidence, cases/100,000 person-years	Incidence rate ratio (95% CI)‡
All reporting ethnicity	255,605	733	36	1 (0.9–1.2)
White				
Any White	111,583 (44)	299	35	Referent
Only White	74,289 (29)	209	38	1.1 (0.9–1.3)
Black	4,925 (2)	5	ND	ND
American Indian, Aleutian, or Eskimo	5,383 (2)	4	ND	ND
Asian				
Only Asian	85,676 (34)	328	46	1.3 (1.1–1.5)
Any Asian	123,187 (48)	423	41	1.2 (1–1.4)
Filipino	49,869 (20)	155	39	1.1 (0.9–1.4)
Japanese	32,238 (13)	137	46	1.3 (1.1–1.6)
Chinese	17,987 (7)	68	43	1.2 (0.9–1.6)
Korean	5,157 (2)	25	63	1.8 (1.2–2.7)
Other Asian	4,463 (2)	7	ND	ND
Vietnamese	1,893 (1)	9	ND	ND
NHOPI				
Only NHOPI	27,003 (11)	36	17	0.5 (0.3–0.7)
Any NHOPI	66,120 (26)	140	27	0.8 (0.6–0.9)
Pacific Islander	51,861 (20)	114	28	0.8 (0.6–1)
Hawaiian	41,853 (16)	115	32	0.9 (0.7–1.1)
Samoan	5,642 (2)	9	ND	ND
Other	18,830 (7)	34	25	0.7 (0.5–1)

*ND, calculation not done because numbers of cases and follow-up times were too low to estimate incidence; NHOPI, Native Hawaiian and Other Pacific Islander; NTM-PI, nontuberculous mycobacterial pulmonary infection. †Ethnic groups are not mutually exclusive except noted by “only.” Ethnicity not reported by 43,218 (14%), single ethnicity reported by 177,016 (69%), multiple ethnicities reported by 78,589 (31%). Only ethnicities for which NTM-PI prevalence was >1% are shown. Asian not presented: Laotian, Asian Indian or Pakistani, Hmong, Kampuchean, Thai; NHOPI not presented: Fiji Islander, Micronesian, Chamorran, Guamanian, Polynesian, Tahitian, Tongan, Melanesian, New Guinean. ‡Incidence rate ratio only calculated for ethnic groups with >10 NTM-PI cases.

**Table 2:** Demographic characteristics for Kaiser Permanente Hawaii beneficiaries, by nontuberculous mycobacterial pulmonary infection status, Hawaii, USA, 2005–2019*

Variable	All, no. (%) or median (IQR)	Probable cases, no. (%) or median (IQR)	Confirmed cases, no. (%) or median (IQR)	Confirmed and probable cases, no. (%) or median (IQR)
Total	298,823	283	456	739
Age, y	42 (30–55)	61 (51–71)	63 (55–71)	63 (54–71)
Sex				
F	148,328 (50)	145 (51)	251 (55)	396 (54)
M	150,495 (50)	138 (49	205 (45)	343 (46)
Asian				
Any	123,187 (48)	150 (54)	273 (60)	423 (58)
Only	85,676 (34)	116 (42)	212 (47)	328 (45)
White				
Any	111,583 (44)	114 (41)	185 (41)	299 (41)
Only	74,289 (29)	81 (29)	128 (28)	209 (29)
NHOPI				
Any	66,120 (26)	62 (22)	78 (17)	140 (19)
Only	27,003 (11)	18 (6)	18 (4)	36 (5)

*IQR, interquartile range; NHOPI, Native Hawaiian and Other Pacific Islander.

### Microbiology and Case Definitions

We queried the KPH EHRs to identify beneficiaries who had undergone mycobacterial testing and had positive NTM culture results. Mycobacterial testing was conducted in a KPH Clinical Laboratory Improvement Amendments–certified laboratory; no methods were changed during the study period. We identified *Mycobacterium avium* complex by using commercially available probes and sent other isolates to Associated Regional and University Pathologists Laboratories (Salt Lake City, UT, USA) for speciation.

We defined cases by using the 2020 American Thoracic Society (ATS) microbiological criteria (*13*). A confirmed case of NTM-PI was defined as either >2 sputum cultures positive for the same pathogenic NTM species or >1 bronchoalveolar lavage, lung biopsy, or pleural fluid cultures positive for the same NTM species. Probable NTM-PI was defined as a single positive sputum culture. We excluded samples from nonpulmonary body sites, *Mycobacterium gordonae*, and samples not identified to complex or species. We included only cultures from beneficiaries who were residents of Hawaii (as determined by 2010 census postal code tabulation areas) for >1 year before the year of culture collection and residents of Hawaii during the year in which the culture was collected.

### Analyses

We included beneficiaries who were >18 years of age at study entry, defined as the first year during the study period when the person had been a KPH beneficiary for >9 months. In addition, we included only beneficiaries who were Hawaii residents for >2 years during the study period. Self-reported ethnic group was termed “only” when a participant reported identification with either Asian, White, or NHOPI groups exclusively and “any” when a participant reported identification with any of those 3 ethnic groups (Table 1). For analysis of BMI, beneficiaries were categorized as underweight (<18.5 kg/m^2^), normal weight (18.5 to <25 kg/m^2^), or overweight (≥25 kg/m^2^) according to their first available BMI score during the study period within 2 years of study entry.

An incident NTM-PI case-patient was defined as a person with an eligible culture and no respiratory cultures positive for NTM (excluding *M. gordonae*) in the prior year. We estimated NTM-PI incidence and compared it across ethnic groups. We described concurrent conditions of interest, including pulmonary conditions and immune disorders, by NTM-PI status and ethnicity. We calculated BMI distribution by ethnic group as well as NTM-PI incidence by BMI and ethnicity. We further described NTM species distribution for persons with confirmed and probable incident NTM-PI.

Our primary analysis was time to incident NTM-PI. We modeled NTM-PI incidence by using multivariable Cox proportional hazards regression. We used patient age as the time scale (*14*), starting with patient’s age at study entry and ending with age in 2019 or the last year in which the person was a KPH beneficiary during the study period. We used 2 approaches to categorize ethnic identification for the purpose of comparison across groups and for modeling disease incidence. For our first approach, we restricted models to beneficiaries self-identifying as only Asian, only White, and only NHOPI. We then modeled ethnic group as a categorical variable with 3 levels (only Asian, only White, or only NHOPI). For our second approach, we restricted the model to beneficiaries who reported ethnicity and modeled self-identifying as Asian by using a single referent group: any Asian versus non-Asian. We modeled Asian subgroups where sample size permitted; Japanese, Chinese, South Korean, and Filipino were modeled as any identification versus no identification. We did not further disaggregate NHOPI subgroups because of small sample size.

We first evaluated univariable models with factors known to be associated with NTM, including sex, BMI, concurrent conditions, and socioeconomic status. We excluded beneficiaries who did not have a BMI measurement within 2 years from the start of follow-up or who did not report ethnicity. On the basis of statistical significance in univariable models and judgment of clinically important confounders, we then constructed a multivariable base model encompassing sex, BMI, pulmonary conditions (modeled as a single binary variable indicating the presence of chronic obstructive pulmonary disease, emphysema, chronic asthma, chronic bronchitis, idiopathic pulmonary fibrosis, hypersensitivity pneumonia, or other unspecified lung diseases, because of high levels of concurrence), immune mechanism disorders, and lung cancer.

We investigated potential interactions between sex and covariates included in the base model and identified a statistically significant interaction between BMI score and sex; for this reason, we then added an interaction term for BMI and sex to the model. We evaluated ethnicity in univariable models and found that ethnic group was associated with NTM risk. When added to the multivariable base model, ethnic group improved model fit as evaluated by likelihood ratio tests. We also evaluated interactions between covariates and ethnicity by constructing a model with interaction terms for ethnicity (only Asian, only White, only NHOPI) and all covariates and compared with likelihood ratio tests for the nested model. We found no statistically significant interactions by ethnicity.

## Results

A total of 298,823 KPH beneficiaries met our analysis inclusion criteria and were considered our population at risk (Table 2). Of those, 739 were classified as NTM-PI case-patients, of which 456 (62%) were confirmed case-patients, resulting in a cumulative incidence of 247 cases/100,000 beneficiaries. The average annual NTM-PI incidence was 44.8 cases/100,000 beneficiaries.

The most commonly reported ethnicity was Asian (123,187 [48%]), followed by White (111,583 [44%]) and NHOPI (66,120 [26%]) (Table 1). Overall, 31% of beneficiaries reported identification with >1 ethnic group. The highest incidence of NTM-PI was 46 cases/100,000 person-years among beneficiaries who self-identified with only the 11 Asian ethnic groups; within the Asian category, the highest incidence was among beneficiaries who self-identified as South Korean (63/100,000 person-years) or Japanese (46/100,000 person-years). The lowest incidence of 17/100,000 person-years was observed among beneficiaries who reported only NHOPI identification, and incidence was similar among NHOPI subgroups.

We compared the demographic and ethnic distribution across categories of probable and confirmed cases (Table 2). Median age was similar across case categories: 61 (interquartile range [IQR] 51–71) years for probable case-patients and 63 (IQR 55–71) years for confirmed case-patients. The distribution of probable and confirmed case-patients was similar across ethnic groups. The census tract median household income was similar for the any White and any NHOPI groups but was somewhat higher for the any Asian group (Table 3). However, the deprivation index was higher for the any NHOPI group compared with the any Asian or any White groups. With respect to underlying conditions, the proportion of beneficiaries with diabetes was highest (24%) among any NHOPI compared with 21% among any Asian and 13% among any White. Prevalence of other concurrent conditions was low; overall rates were similar among the ethnic groups. Median age varied by ethnic group; the NHOPI population was younger on average (median age 37 years) relative to the Asian population (median age 42 years) and the White population (median age 45 years).

**Table 3:** Clinical and demographic features for Kaiser Permanente Hawaii beneficiaries, by ethnic group, Hawaii, USA, 2005–2019*

Variable	Any Asian, no. (%) or median (IQR)	Any White, no. (%) or median (IQR)	Any NHOPI, no. (%) or median (IQR)
Age, y	42 (30–56)	45 (32–57)	37 (27–50)
Ever smoked	32,375 (28)	32,577 (31)	22,612 (37)
Census-tract socioeconomic measures			
Household income, USD	72,634 (58,184–88,143)	68,359 (58,295–83,750)	68,359 (54,470–81,464)
Neighborhood deprivation index	−0.23 (−0.62 to 0.28)	−0.33 (–0.65 to 0.07)	−0.08 (–0.48 to 0.51)
Graduation from high school	90 (84–94)	92 (88–95)	90 (85–93)
BMI			
BMI, kg/m^2^	25.9 (22.7–30)	26.6 (23.3–31)	30.5 (25.9–35.9)
Underweight, <18.5 kg/m^2^	2,853 (3)	1,725 (2)	531 (1)
Normal weight, 18.5 to <25, kg/m^2^	41,699 (40)	35,606 (36)	10,637 (19)
Overweight or obese, >25 kg/m^2^	60,551 (58)	62,107 (62)	44,890 (80)
Concurrent conditions			
Diabetes	25,676 (21)	14,385 (13)	15,543 (24)
Lung cancer	1,083 (1)	1,099 (1)	582 (1)
Bronchiectasis	842 (1)	518 (<1)	293 (<1)
Chronic asthma	2,726 (2)	2,795 (3)	2,044 (3)
Chronic obstructive pulmonary disease	4,788 (4)	5,709 (5)	3,004 (5)
Emphysema	1,093 (1)	1,357 (1)	630 (1)
Chronic bronchitis	1,993 (2)	2,323 (2)	1,436 (2)
Other pulmonary disease	3,099 (3)	3,319 (3)	1,747 (3)
Immune mechanism disease	457 (<1)	586 (1)	225 (<1)

*BMI, body mass index; IQR, interquartile range; NHOPI, Native Hawaiian and Other Pacific Islander.

Few KPH beneficiaries were categorized as underweight (5,027 [2%]); most were in the overweight/obese clinical category (149,970 [62%]). BMI varied by ethnic group; the highest proportion of persons with normal or underweight BMI scores was among persons self-identifying as Asian (44,552 [43%]), and a lower proportion of persons with normal or underweight BMI scores was among persons who identified as NHOPI (11,168 [20%]) (Table 3). In contrast, among NTM-PI case-patients, 45 (6.3%) were underweight, 324 (45.4%) were normal weight, and 345 (48.3%) were overweight/obese. Across all ethnic groups, the pattern of increasing NTM-PI incidence with decreasing BMI was similar (Figure); compared with beneficiaries who were normal weight, beneficiaries who were underweight had a 1.4- to 2.5-fold increased risk for NTM-PI. The NTM-PI risk among beneficiaries who were normal weight compared with those who were overweight was also 1.4- to 1.8-fold higher.

**Figure:**
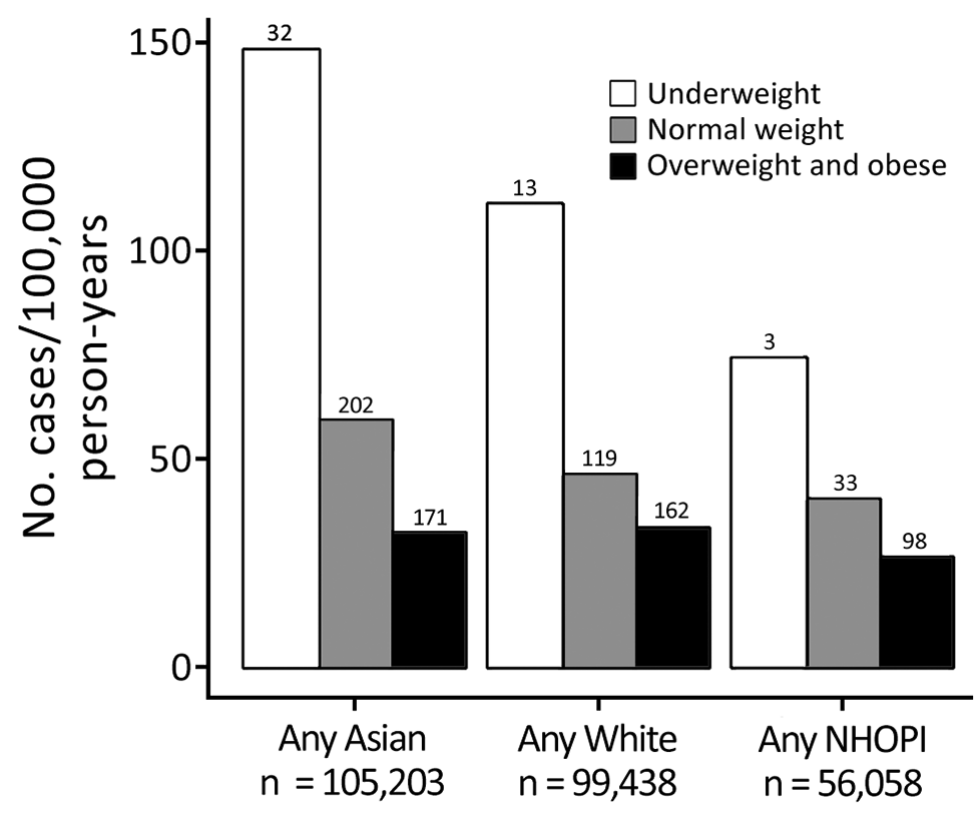
Nontuberculous mycobacterial pulmonary infection incidence among Kaiser Permanente Hawaii beneficiaries, by ethnicity and body mass index category, Hawaii, USA, 2005–2019. Numbers above bars indicate incidence (cases/100,000 person-years) by BMI category. Underweight, <18.5 kg/m^2^; normal weight, 18.5 to <25 kg/m^2^; overweight/obese, >25 kg/m^2^. NHOPI, Native Hawaiian and Other Pacific Islander.

To assess the effect of BMI change on NTM-PI risk, we evaluated change in weight among NTM-PI case-patients and non–NTM-PI controls. In the 2-year period (±6 months) before incident culture collection, the weight of NTM-PI case-patients decreased; median change was −1.9% (IQR −6.7% to 1.5%). A convenience sample of non–NTM-PI controls included beneficiaries >53 years of age (the first quartile age of NTM-PI case-patients), whose first and last weight measurements were 2 years (±6 months) apart. Median weight change for controls was generally stable, showing a slight decrease of −0.25% (IQR −4.2% to 3.3%).

When we evaluated the frequency of microbiological follow-up among case-patients, the median number of acid-fast bacteria cultures performed during the study period was 4 (IQR 3–7); range was 1–43. The median follow-up time was 6 years (IQR 3–12 years). Most case-patients had only 1 incident NTM-PI (608 [82%]), but 131 (18%) had >2 incident infections. The most common NTM species isolated was *M. avium* complex (69%), followed by *M. fortuitum* group (24%) and *M. abscessus* (21%) (Appendix Table 2). We found little difference in *Mycobacterium* spp. distribution among ethnic groups (Appendix Table 3). We further evaluated our microbiological case definition relative to the ICD-9/10 code for NTM-PD (ICD-9, 031.0; ICD-10, A31.0) and found 69% sensitivity for confirmed cases, 25% sensitivity for probable cases, >99% specificity, and a positive predictive value of 73%.

In models in which ethnicity was a mutually exclusive categorical variable with only White as the reference group, the risk for incident NTM-PI was increased for the only Asian group (30%) (adjusted hazard ratio [aHR] 1.3, 95% CI 1.1–1.6). For the only NHOPI group relative to the only White group, the aHR for NTM-PI did not differ significantly (aHR 0.92, 95% CI 0.63–1.3) (Table 4). The aHR was similar when ethnicity was modeled as a binary variable indicating whether the beneficiary self-identified as any Asian or no Asian; risk for NTM-PI was increased 30% for those with identification as any Asian relative to no Asian (aHR 1.3, 95% CI 1.1–1.5). Analysis of Asian subgroups indicated a stronger association for Filipinos; NTM-PI risk was higher among those self-identifying as Filipino versus not Filipino than among those in the broader any Asian category. The association between BMI and NTM-PI incidence varied by sex. Decreasing BMI was associated with a higher NTM-PI risk for women than for men. In multivariable models that also included concurrent conditions and ethnicity and were controlled for age, each 1 kg/m^2^ increase in BMI decreased the risk for infection by 9% for women and 4% for men.

**Table 4:** Cox proportional hazards regression of risk for NTM-PI among Kaiser Permanente Hawaii beneficiaries, Hawaii, USA, 2005–2019*

Category	Only White reference, aHR (95% CI)†	Only Asian reference, aHR (95% CI)†	Not Asian reference, aHR (95% CI)‡
Body mass index§			
M	0.96 (0.94–0.99)	0.96 (0.94–0.99)	0.97 (0.95–0.99)
F	0.91 (0.89–0.93)	0.91 (0.89–0.93)	0.91 (0.89–0.93)
Ethnicity			
Only White	Referent	0.77 (0.64–0.92)	NA
Only Native Hawaiian and Other Pacific Islander	0.9 (0.62–1.3)	0.69 (0.48–1)	NA
Only Asian	1.3 (1.1–1.6)	Reference	NA
Any Asian	NA	NA	1.3 (1.1–1.5)

*Models adjusted for pulmonary condition (chronic obstructive pulmonary disease, chronic asthma, emphysema, chronic bronchitis, idiopathic pulmonary fibrosis, hypersensitivity pneumonia, other unclassified lung diseases); malignant neoplasm of trachea, bronchus, and lung; disorders involving the immune mechanism. aHR, adjusted hazard ratio; NA, not applicable; NTM-PI, nontuberculous mycobacterial pulmonary infections. †No. modeled = 161,465; no. NTM-PI cases = 554. ‡No. modeled = 220,493; no. NTM-PI cases = 709. §No. missing: only Asian and only White reference = 25,503; not Asian reference = 35,112.

## Discussion

We found that within the state of Hawaii, risk for NTM-PI was increased among persons who self-identified with any Asian ethnicity, even after we controlled for demographic and clinical risk factors. In contrast, after adjusting for these same demographic and clinical risk factors, risk was similar for persons who self-identified as any NHOPI compared with persons who self-identified as any White. A unique aspect of our study is that we have detailed information on Asian subgroups; the group categorized in Hawaii as Asian represents populations originating in several countries, primarily Japan, China, South Korea, and the Philippines. The higher risk for NTM-PI among persons identifying with Asian subgroups in our study is consistent with available data from countries in northeast Asia. Those data indicate generally higher disease burdens in Japan, South Korea, and Taiwan compared with estimates from Europe and other parts of the United States (*15*). Limited to no data are available from the Philippines or other countries in Southeast Asia.

A recent population-based study from South Korea estimated an age-adjusted incidence of 17.9 cases/100,000 persons and prevalence of 33.3 cases/100,000 persons for 2016 (*16*). In Japan, a hospital survey–based NTM-PD estimate was 15 cases/100,000 persons in 2015 (*17*). In the US-Affiliated Pacific Islands, a laboratory-based study estimated an NTM-PI prevalence of 48 cases/100,000 persons in 2011; prevalence increased during 2007–2011 (*8*), similar to the average annual NTM-PI incidence of 44.8 cases/100,000 beneficiaries estimated in our study. Average annual NTM-PI incidence in our study is probably higher than that estimated in Japan and South Korea because of their more specific case criteria, which are based on full ATS NTM-PD diagnosis guidelines.

The reasons for the increased risk for Asian populations, those resident in Hawaii as well as in Asian countries, are not clear. Higher risk associated with self-described ethnicity may represent a mix of genetic ancestry, behavioral, or environmental factors. Because we did not measure specific behaviors or exposures and do not have any precise measures of genetic admixtures, we cannot estimate the relative contribution of these factors to our findings. Findings from a multiethnic cohort study (a large, ongoing, prospective study of diet, lifestyle, and genetic risks) suggests a role for differences in susceptibility among populations identifying with East Asian and Native Hawaiian ethnicity, even after lifestyle factors were accounted for (*18*).

Because exposure to nontuberculous mycobacteria is common, particularly in a high-risk geographic area such as Hawaii (*19*), but disease is still relatively rare, host susceptibility with involvement of multiple genes and pathways probably plays a role in pathogenesis (*20*). Several studies have evaluated the role of genetics among persons of various ethnic groups and used a variety of approaches, including candidate gene approaches (*21*), whole-exome sequencing (*22*), and genome-wide association studies (*23*,*24*). Most recently, genome-wide association studies in Japan and South Korea have identified single-nucleotide polymorphisms (SNPs) associated with disease susceptibility. However, the relative prevalence of some of those SNPs in different populations remain unknown, and further studies are needed to compare the prevalence of these SNPs across populations.

We found an inverse relationship between BMI and NTM risk. Risk was highest for clinically underweight beneficiaries compared with those with BMIs categorized as normal or overweight/obese. These findings of an increased risk for incident NTM with decreasing BMI is consistent with findings of other studies showing not only the role of low BMI in disease progression (*25*,*26*) but also in susceptibility to infection or disease. A recent study that used South Korea national insurance data and prospectively ascertained BMI and NTM-PD over 9 years (*27*) found that lower BMI at baseline as well as weight loss during the study period were associated with higher NTM-PD risk. Proposed mechanisms for increased risk among persons with low BMI include fat loss with changes in adipokines (e.g., as leptin, resistin, and adiponectin) (*28*). Studies of mice have shown that experimental fat ablation can contribute to increased lung disease during mycobacterial infection (*29*). Leptin levels decreased with lower BMI, and pulmonary bacterial loads after *Mycobacterium tuberculosis* infection in mice that were genetically deficient in leptin were higher (*30*). Nevertheless, the specific effects of individual adipokines on host resistance to mycobacterial NTM in human infection remain unknown (*28*). The association between BMI and NTM-PI depended on sex in our study population, in which decreasing BMI was associated with proportionally higher risk among women than men. It is hypothesized that estrogen, leptin, and adiponectin may play an additional role in susceptibility to NTM-PD in older women (*31*).

A strength of our study is the large sample size that included persons of diverse Asian and Pacific Islander ethnicities in a single geographic area where risk for NTM infection is high. Among the limitations of our study was our reliance on the microbiological component of the ATS case criteria because we did not have radiographic or symptom data (*13*); however, other studies indicate that the microbiological component predictive value for true cases is high. In a similar retrospective study at an integrated healthcare system, among persons from whom >1 NTM isolate was obtained, 69.5% had nodules, bronchiectasis, or cavities compatible with NTM disease (*32*). Moreover, the finding that the 69% of those with confirmed cases and the 25% of those with probable cases had ICD-9/10 codes, with an overall positive predictive value of 73%, suggests that we are identifying a high proportion of true disease, particularly among persons with confirmed cases.

A second limitation is that follow-up was not standardized in this beneficiary population, and therefore the availability of BMI measurements was associated with healthcare use. Overall, 33,269 (11%) beneficiaries did not have a BMI measurement available; those who did not have a BMI measurement were more likely to be younger and to not have any underlying conditions. However, we have no evidence that these missing data influenced the patterns by ethnic group. Use of BMI score as a measurement of body composition is itself a limitation because it does not distinguish fat mass from muscle mass. Last, ethnicity groupings are artificial, given that populations in Hawaii are highly heterogenous and 31% of beneficiaries reported >1 ethnicity. In addition, Asian and NHOPI identification is defined broadly and includes diverse subpopulations. Caution should be taken when generalizing these findings to ethnic populations outside of Hawaii (*9*).

In conclusion, we found that lower BMI was associated with increased risk for NTM-PI and that self-identifying as Asian, independent of other ethnic identification, was associated with a higher risk for NTM-PI. Whereas risk for NTM-PI seemed to be lower for the NHOPI population, our findings suggest that after confounders were controlled for, the risk is similar to that for beneficiaries self-identifying as White. 

Appendix Supplemental results for study of incidence of nontuberculous mycobacterial pulmonary infection, by ethnic group, Hawaii, USA, 2005–2019.
