# Correction: Vol. 28, No. 8

**DOI:** 10.3201/eid2810.C22810

**Published:** 2022-10

**Keywords:** errata, erratum, correction

The Figure legend has been corrected to refer to case counts by category in Incidence of Nontuberculous Mycobacterial Pulmonary Infection, by Ethnic Group, Hawaii, USA, 2005–2019 (R.A. Blakney et al.). The article has been corrected online (https://wwwnc.cdc.gov/EID/article/28/8/21-2375_article).

