# Supplementary material for: Incidence of Nontuberculous Mycobacterial Pulmonary Infection, by Ethnic Group, Hawaii, USA, 2005–2019
Source: Emerg Infect Dis. 2022 Aug;28(8):1543–50. doi: 10.3201/eid2808.212375 (PMC9328927; doi:10.3201/eid2808.212375)
Supplement: Appendix — Supplemental results for study of incidence of nontuberculous mycobacterial pulmonary infection, by ethnic group, Hawaii, USA, 2005–2019. [file 21-2375-Techapp-s1.pdf]

Article DOI: https://doi.org/10.3201/eid2808.212375

Incidence of Nontuberculous Mycobacterial Pulmonary Infection, by Ethnic Group, Hawaii, USA, 2005–2019

Appendix

Appendix Table 1. Comparison of Kaiser Permanente (KPH) study population to Hawaii census data, 2019

Variable	Census (%)	KPH (%)
Age (years)		
< 18	21	18
18-39	29	29
40-59	24	28
60-79	20	21
> 80	5	3
Ethnicity		
Only white (%)	26	23
Only Asian	38	33
Only Native Hawaiian and Other Pacific Islander	10	12
Highschool graduation (%)*	92	91
Median household income ($)*	81,275	70,909

*Median.

Appendix Table 2. Distribution of NTM species by case status, Kaiser Permanente Hawaii, 2005-2019

Species*	Confirmed cases, no. (%)	Probable cases, no. (%)	Confirmed and probable cases, no. (%)
M. avium complex	308 (68)	205 (72)	513 (69)
M. abscessus	118 (26)	40 (14)	158 (21)
M. fortuitum group	87 (19)	88 (31)	175 (24)
M. kansasii	6 (1)	6 (2)	12 (2)
Other rapid grower	6 (1)	5 (2)	11 (1)
Other slow grower	3 (< 1)	9 (3)	12 (2)

**M. abscessus* includes: *M. abscessus*, *M. chelonae-abscessus* and *M. chelonae*.

Appendix Table 3. Distribution of NTM species by ethnicity, Kaiser Permanente Hawaii, 2005-2019

Species*	Any Asian, no. (%)	Any White, no. (%)	Any Native Hawaiian and Other Pacific Islander, no. (%)
M. avium complex	288 (68)	214 (71)	91 (65)
M. abscessus	101 (24)	57 (19)	21 (15)
M. fortuitum group	104 (25)	72 (24)	37 (26)
M. kansasii	5 (1)	6 (2)	4 (3)
Other rapid grower	7 (2)	2 (1)	1 (1)
Other slow grower	5 (1)	7 (2)	4 (3)

**M. abscessus* includes: *M. abscessus*, *M. chelonae-abscessus* and *M. chelonae*.
